# Supplementary material for: Affordances for throwing: An uncontrolled manifold analysis
Source: PLoS One. 2024 Apr 17;19(4):e0301320. doi: 10.1371/journal.pone.0301320 (PMC11023389; doi:10.1371/journal.pone.0301320)
Supplement: Attachment — Submitted filename: Bennett et al. Plos One Review.docx [file pone.0301320.s001.docx]

**Affordance for Throwing: An Uncontrolled Manifold Analysis**

Thank you for the opportunity to review the manuscript. The manuscript is well written and provides a first attempt to ground typically unconstrained analyses—such as UCM—within the theoretical realm of affordances. With a few edits mainly related to producing an easier to read and succinct paper, I believe this is an excellent manuscript that contributes to both UCM and affordance literature. My specific comments are listed below:

**Specific Comments**

**Introduction**:

In general, I think the introduction could be shortened up. For, example you mention the manuscript’s hypotheses in the “Affordances as Dynamical Properties of Tasks” subsection and then hint around them again in the “Current Study” section. You also mention the results of the study on page 5. This is unnecessary in my opinion and leads to a.

First Paragraph—it is awkward to read “another way to phrase this” twice in a row. Maybe you can replace one with “said differently” or something similar?

You defined UCM very early on in the introduction but consistently write out uncontrolled manifold analysis (UCM) throughout the remainder of the manuscript. Please either use “uncontrolled manifold” or “UCM”

Is Perspex a brand name or something?

You mention “affordance properties of the environment”. My understanding is that affordances are defined—or exist—relative to the animal-environment properties and that you can’t solely have affordance properties of either the animal or the environment alone. They (i.e., affordances) must be defined relative to the combination of animal/environment properties. I may be misunderstanding, but I think that is the issue—we don’t have to agree what an affordance is, but I am confused with your use of it, could you please define/operationalize it more clearly?

**Method:**

You mention 10 participants in the participant section but then only list 7 participants (6 right-handed and 1 left-handed) on page 7? Please correct.

In my opinion, you don’t need to list the calibration information for the motion capture system—everyone assumes you have performed that =]

**Results**:

I don’t believe the first paragraph in the Results section belongs there, instead it should go under “Data Analysis” section. Similarly, you should have a statistical analysis section where you list how you performed the stats, this should not in the results section (for example, where you state sphericity is violated—this should be in the method).

Again, you shouldn’t be interpreting the results in the results section (i.e., some of the information on page 21 under “There are several things to note:”. This belongs in the discussion.

**Discussion**:

On page 25 can you clarify if you do in fact mean calibration or do you perhaps mean attunement? However, this is probably a distinction that is beyond the scope of this paper…

Again, I am confused by your use of affordance in the last paragraph on page 25—I thought an affordance is either “existing” or not. If you can’t perform an action, then it can’t possibly be an affordance? Again, we can agree/disagree on what an affordance is, but, at least to me, I’m getting conflicting definitions throughout the manuscript.
