# Supplementary material for: Affordances for throwing: An uncontrolled manifold analysis
Source: PLoS One. 2024 Apr 17;19(4):e0301320. doi: 10.1371/journal.pone.0301320 (PMC11023389; doi:10.1371/journal.pone.0301320)
Supplement: Attachment — Submitted filename: Reply to Reviews - R1.docx [file pone.0301320.s002.docx]

# Reply to Reviews

*Reviewer #1: In my opinion, it is better to fix the structure of the article and the text of the article is very long, and it is better to use a short and summarized text. It is a good article and in my opinion it is suitable for the Journal.*

We have removed ~450 words to remove any repetition, etc. We hope this works for the reviewer; they didn’t have any specific editing requests and we had quite a bit of ground to cover.

*Reviewer #2*

*Thank you for the opportunity to review the manuscript. The manuscript is well written and provides a first attempt to ground typically unconstrained analyses—such as UCM—within the theoretical realm of affordances. With a few edits mainly related to producing an easier to read and succinct paper, I believe this is an excellent manuscript that contributes to both UCM and affordance literature. My specific comments are listed below:*

Thank you for these positive comments.

***Introduction****:*

*In general, I think the introduction could be shortened up. For, example you mention the manuscript’s hypotheses in the “Affordances as Dynamical Properties of Tasks” subsection and then hint around them again in the “Current Study” section. You also mention the results of the study on page 5. This is unnecessary in my opinion and leads to a.*

We have removed all the repetition mentioned here.

*First Paragraph—it is awkward to read “another way to phrase this” twice in a row. Maybe you can replace one with “said differently” or something similar?*

We changed this as part of the editing to just say ‘Formally’ but kept the repetition to provide a rhythm for the reader.

*You defined UCM very early on in the introduction but consistently write out uncontrolled manifold analysis (UCM) throughout the remainder of the manuscript. Please either use “uncontrolled manifold” or “UCM”*

Fixed. We use UCM for the analysis, and ‘uncontrolled manifold’ only when actually talking about the manifold.

*Is Perspex a brand name or something?*

Yes, so the capitalization is standard.

*You mention “affordance properties of the environment”. My understanding is that affordances are defined—or exist—relative to the animal-environment properties and that you can’t solely have affordance properties of either the animal or the environment alone. They (i.e., affordances) must be defined relative to the combination of animal/environment properties. I may be misunderstanding, but I think that is the issue—we don’t have to agree what an affordance is, but I am confused with your use of it, could you please define/operationalize it more clearly?*

We have edited throughout the manuscript to be as consistent and clear as possible. There are two ways to formalize affordances in the literature, as properties of organism-environment systems that reside in the environment part (which we use here) and as relations between organisms and environments (the account the reviewer describes here). We use the former because only this way of formalizing affordances leads to the task-dynamical analysis, and because one of us (ADW) has written extensively criticizing the relational account as insufficient. We have not talked about this debate here because it’s a complicated tangent that would just be distracting here; what we have done is to just be clear which account of affordances we are endorsing and relying on, and made some edits to improve that clarity and consistency. Please let us know whether it’s working!

***Method:***

*You mention 10 participants in the participant section but then only list 7 participants (6 right-handed and 1 left-handed) on page 7? Please correct.*

We noted already that 3 were excluded due to data collection problems; we’ve clarified this.

*In my opinion, you don’t need to list the calibration information for the motion capture system—everyone assumes you have performed that =]*

Removed this section and just noted that the recording volume was a calibrated one.

***Results****:*

*I don’t believe the first paragraph in the Results section belongs there, instead it should go under “Data Analysis” section. Similarly, you should have a statistical analysis section where you list how you performed the stats, this should not in the results section (for example, where you state sphericity is violated—this should be in the method).*

*Again, you shouldn’t be interpreting the results in the results section (i.e., some of the information on page 21 under “There are several things to note:”. This belongs in the discussion.*

We have moved all these sections as suggested, and made a couple of edits just to make them fit in their new location.

***Discussion****:*

*On page 25 can you clarify if you do in fact mean calibration or do you perhaps mean attunement? However, this is probably a distinction that is beyond the scope of this paper…*

Definitely calibration. Ecologically, attunement is about detecting relevant information, while calibration is about scaling the action.

*Again, I am confused by your use of affordance in the last paragraph on page 25—I thought an affordance is either “existing” or not. If you can’t perform an action, then it can’t possibly be an affordance? Again, we can agree/disagree on what an affordance is, but, at least to me, I’m getting conflicting definitions throughout the manuscript.*

Hopefully the effort to be clear and consistent helps here. But just to note: affordances considered as properties of organism-environment systems exist and are on offer, and organisms vary in their ability to effect those affordances; this is necessary in order to be able to learn to effect new affordances. Getting into the specific to justify this here would again require spending a lot of time on the details of that debate. We’ve chosen not to given that both reviewers wanted the paper to be shorter and that this paper isn’t about that debate; the goal is to be doing empirical work specifically motivated by the properties account of affordances.

Again, though, please let us know whether we have been clear and consistent here though.
